# Comparisons of Drug-Eluting Balloon versus Drug-Eluting Stent in the Treatment of Young Patients with Acute Myocardial Infarction

**DOI:** 10.3390/jcdd10010029

**Published:** 2023-01-13

**Authors:** Yi-Xing Yang, Kui-Zheng He, Jiang-Yuan Li, Yuan Fu, Chuang Li, Xin-Ming Liu, Hong-Jiang Wang, Mu-Lei Chen, Pi-Xiong Su, Li Xu, Le-Feng Wang

**Affiliations:** Heart Center and Beijing Key Laboratory of Hypertension, Beijing Chaoyang Hospital, Capital Medical University, No. 8, Gongti South Road, Chaoyang District, Beijing 100020, China

**Keywords:** drug-eluting balloon, acute myocardial infarction, young patients

## Abstract

Background: The incidence of acute myocardial infarction (AMI) in the younger population has been increasing gradually in recent years. The objective of the present study is to investigate the safety and effectiveness of drug-eluting balloons (DEBs) in young patients with AMI. Methods: All consecutive patients with AMI aged ≤ 45 years were retrospectively enrolled. The primary endpoint was a device-oriented composite endpoint (DOCE) of cardiac death, target vessel myocardial infarction (MI), or target lesion revascularization (TLR). The secondary study endpoints included heart failure and major bleeding events. Results: A total of 276 young patients presenting with AMI were finally included. The median follow-up period was 1155 days. Patients treated with DEBs had a trend toward a lower incidence of DOCEs (3.0% vs. 11.0%, *p* = 0.12) mainly driven by the need for TLR (3.0% vs. 9.1%, *p* = 0.19) than those treated with DESs. No significant differences between the two groups were detected in the occurrence of cardiac death (0.0% vs. 0.5%, *p* = 0.69), MI (0.0% vs. 1.4%, *p* = 0.40), heart failure (0.0% vs. 1.9%, *p* = 0.39), or major bleeding events (1.5% vs 4.8%, *p* = 0.30). Multivariate regression analysis showed that DEBs were associated with a trend toward a lower risk of DOCEs (HR 0.13, 95% CI [0.02, 1.05], *p* = 0.06). Conclusions: The findings of the present study suggested that DEBs might be a potential treatment option in young patients with AMI. A larger scale, randomized, multicenter study is required to investigate the safety and effectiveness of DEBs in this setting.

## 1. Introduction

Although the incidence of acute myocardial infarction (AMI) in the general population has significantly declined during the last decades, this decreasing tendency has not been uniformly observed in the young population [1]. In fact, the proportion of AMI hospitalizations attributable to younger individuals has been increasing gradually in recent years and accounts for up to 4–10% of all AMIs [2,3]. Moreover, the long-term prognosis in the young population is unfavorable, as it indicates that the death rate is up to 12–20%, and the rate of major adverse cardiac events (MACE) reaches 23–30% during the 15-year follow-up period [4,5,6]. Additionally, AMI occurring at a young age carries significant morbidity, psychological consequences, and economic burdens for patients and families because it affects those who are in the stage of full productivity [7,8]. Therefore, there is a great need to advance more effective awareness and treatment strategies specific to this important clinical entity.

According to current guidelines, percutaneous coronary intervention (PCI) with a drug-eluting stent (DES) is the preferred revascularization strategy for patients of all ages who present with AMI for the benefit of timely recovering coronary artery blood flow as well as decreasing the risk of repeat revascularization [9]. However, permanent vascular implants after implanting DES could lead to an increased risk of late and very late stent thrombosis, and recent studies suggested that younger age was an independent risk factor for late stent thrombosis (LST) and very late stent thrombosis (VLST) [10,11,12]. Additionally, intervention with a stent has been demonstrated to be associated with increased stress disorder in younger individuals [13]. Therefore, the concept of avoiding permanent implants may be especially attractive for younger patients with AMI.

A drug-eluting balloon (DEB) is a novel treatment strategy that has emerged in recent years and has been proven to be safe and effective in treating patients with in-stent restenosis and shown promising results in other indications such as small vessel disease, diffuse disease, bifurcations, chronic total occlusions, and calcified complex lesions [14,15]. The advantage of DEB is that it could rapidly provide a homogeneous distribution and high-concentration of antirestenotic drugs into the target lesion of the culprit coronary artery without using durable polymers and stent structures, thus fulfilling the requirements of “leaving nothing behind” to avoid long-term, stent-related complications as well as reduce the risk of stress disorders after PCI. However, to date, there are no data investigating the safety and effectiveness of DEBs among young patients with AMI. The objective of the present study is to investigate the safety and effectiveness of DEBs compared with DESs for young patients presenting with AMI.

## 2. Methods

### 2.1. Study Design and Population

All consecutive patients admitted to Beijing Chaoyang Hospital with AMI from 1 January 2017 to 1 January 2022 were retrospectively analyzed in this single-center study. Patients were included if they met the following criteria: (1) age 18 to 45 years; (2) presenting with ST-segment elevation myocardial infarction (STEMI) undergoing primary PCI or presenting with non-ST-segment elevation myocardial infarction (NSTEMI) undergoing early invasive strategy (<24 h from symptom onset); and (3) receiving DES or DEB treatment. Patients were excluded if they met the following criteria: (1) presenting with stable or unstable angina pectoris; (2) cardiac arrest or cardiogenic shock; (3) mechanical complications; (4) ongoing malignant process; (5) receiving conservative treatment; (6) in-stent restenosis; (7) severe coronary artery tortuosity and calcification; (8) coronary artery ectasia; (9) undergoing plain old balloon angioplasty (POBA), a bare metal stent (BMS), or coronary bypass surgery; or (10) the combination use of DES and DEB in the target vessel segment. This study complied with the Declaration of Helsinki and has been approved by the ethical committee of Beijing Chaoyang Hospital.

### 2.2. Interventional Procedure

The PCI procedure was performed according to current international guidelines and local practice. All patients were administered 300 mg of aspirin and 300–600 mg of clopidogrel or 180 mg of ticagrelor as loading doses before the procedure. Unfractionated heparin was intravenously given as an initial bolus of 100 IU/kg body weight followed by additional boluses to maintain an activated clotting time of ≥250 s during the procedure.

In all cases, decisions regarding intervention strategy (DES or DEB), appropriate length and diameter of DES or DEB, inflation time and pressure of DES or DEB, administration of intra-aortic balloon pump, thrombus aspiration, and glycoprotein IIb/IIIa inhibitor during the procedure were left to the discretion of the operator. The procedure was considered successful if the visual postprocedural residual stenosis was ≤30% after PCI. A bailout stenting was performed if there was an apparent flow-limiting dissection (grade C–F) or residual stenosis >30% after DEB implantation.

After PCI, a dual antiplatelet therapy (DAPT) was prescribed using aspirin (100 mg/d) and either clopidogrel (75 mg/d) or ticagrelor (90 mg twice per day). DAPT was suggested for 3–6 months after DCB and 12 months in patients treated with DES.

### 2.3. Endpoints and Definitions

The primary endpoint was a device-oriented composite endpoint (DOCE) of cardiac death, target vessel myocardial infarction (MI), or target lesion revascularization (TLR) [16]. All deaths were considered cardiac unless a definite noncardiac cause could be documented. MI was defined as recurrent ischemic symptoms lasting ≥30 min together with either new electrocardiographic changes or elevation of troponin or creatine kinase MB isoenzyme level. Target vessel MI was defined as an MI not clearly attributable to a nontarget vessel. TLR was defined as any repeat percutaneous intervention or bypass surgery of the target lesion (including the whole DEB- or DES-treated segment plus 5 mm proximal and distal of the treated segment) due to restenosis, stent thrombosis, and coronary dissection. The secondary study endpoints included heart failure and major bleeding events. Heart failure was defined as any congestive heart failure (rales, dyspnoea, and New York Heart Association (NYHA) class III–IV) after the index procedure [17]. Major bleeding events were defined according to the International Society on Thrombosis and Hemostasis (ISTH) criteria [18].

### 2.4. Clinical Follow-Up

Clinical follow-up by reviewing hospital records, clinic visits, and telephone interviews was conducted during hospitalization and at 30 days, 3 months, 6 months, and 12 months after the index procedure and every three months thereafter. All adverse events were adjudicated by independent physicians who were not involved in the procedures.

### 2.5. Statistical Analysis

Continuous data were expressed as mean ± SD or median and interquartile range and were compared using Student’s t-test or Mann–Whitney rank-sum test. Categorical data were expressed as numbers and percentages and compared using Pearson’s chi-square test or Fisher’s exact test. Event-free survival was estimated with the Kaplan–Meier method and compared using the log-rank test. Multivariate Cox proportional hazard regression models were performed to identify significant independent predictors of the endpoints. Prespecified subgroup analyses by age (≤40 vs. >40 years), infarct-related artery (left anterior descending artery vs. nonleft anterior descending artery), Killip class (Killip class I vs. II–III), thrombolysis in myocardial infarction (TIMI) flow pre-PCI (0–1 vs. 2–3), device diameter (<3 vs. ≥3 mm), device length (<30 vs. ≥30 mm), non-infarct-related artery stenosis (<70% vs. ≥70%), the use of thrombus aspiration, and additional administration of glycoprotein IIb/IIIa inhibitors were performed to evaluate the consistency of treatment effects. Interactions between treatment groups were analyzed using Cox proportional hazards models. A value of *p* < 0.05 was considered statistically significant. All statistical analyses were performed using SPSS 26.0 software (SPPS Inc., Chicago, IL, USA).

## 3. Results

**Figure 1:**
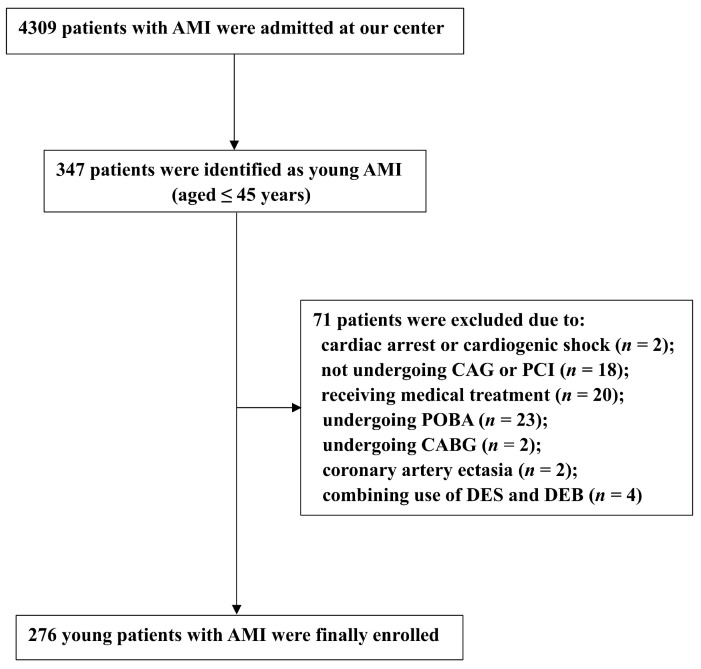
Patient Flow Chart. AMI: acute myocardial infarction; CAG: coronary angiography; PCI: percutaneous coronary intervention; POBA: plain old balloon angioplasty; CABG: coronary bypass surgery; DEB: drug-eluting balloon; DES: drug-eluting stent.

From January 2017 to January 2022, a total of 4309 patients with AMI were admitted to Beijing Chaoyang Hospital, and 347 of them were identified as young AMI patients who were at or under 45 years old. Among these, 71 patients were not eligible for this study due to the following reasons: cardiac arrest or cardiogenic shock (*n* = 2); not undergoing coronary angiography or PCI (*n* = 18); receiving medical treatment (*n* = 20); undergoing plain balloon angioplasty (*n* = 23); undergoing coronary bypass surgery (*n* = 2); coronary artery ectasia (*n* = 2); or the combination use of DES and DEB in the target vessel segment (*n* = 4). Thus, a total of 276 younger patients presenting with AMI were enrolled in the final analysis, including 67 patients receiving DEB treatment and 209 patients receiving DES treatment (Figure 1).

Baseline demographic, clinical, laboratory, and angiographic characteristics are shown in Table 1 and Table 2. The mean age of the overall population was 39.3 ± 4.1 years, and 94.9% of them were men. Smoking (81.9%) was found to be the major risk factor for young AMI patients followed by overweightness (76.8%), hypertension (45.7%), family history of coronary artery disease (44.2%), hyperlipidemia (22.5%), and diabetes mellitus (18.5%). The majority (62.3%) of the young patients presented with STEMI. Multiple vessel disease (non-infarct-related artery with stenosis >70%) was observed in 63.0% of patients, and the left anterior descending coronary artery (LAD) was the most frequent infarct-related artery (41.7%).

Compared with patients treated with DESs, patients treated with DEBs had a lower prevalence of STEMI (47.8% vs. 67.0%, *p* = 0.01), fewer LAD lesions as an infarcted related artery (28.4% vs. 45.9, *p* = 0.01), smaller device number (1.0 [1.0, 1.0] vs. 2.0 [1.0, 2.0], *p* < 0.01), smaller device diameter (2.75 vs. 3.00, *p* < 0.01), shorter device length (26.00 [20.00, 30.00] vs. 30.00 [23.5, 46.00], *p* < 0.01), lower inflation pressure (9.31 ± 1.84 vs. 10.58 ± 1.76, *p* < 0.01), and lower frequency of using thrombus aspiration (9.0% vs. 23.0%, *p* = 0.01). Moreover, among those patients who had non-infarct-related arteries with stenosis > 70%, the rate of immediate complete revascularization was higher in the DEB group than in the DES group (53.2% vs. 22.8%, *p* < 0.01). No significant differences were observed with regard to other baseline characteristics.

**Figure 2:**
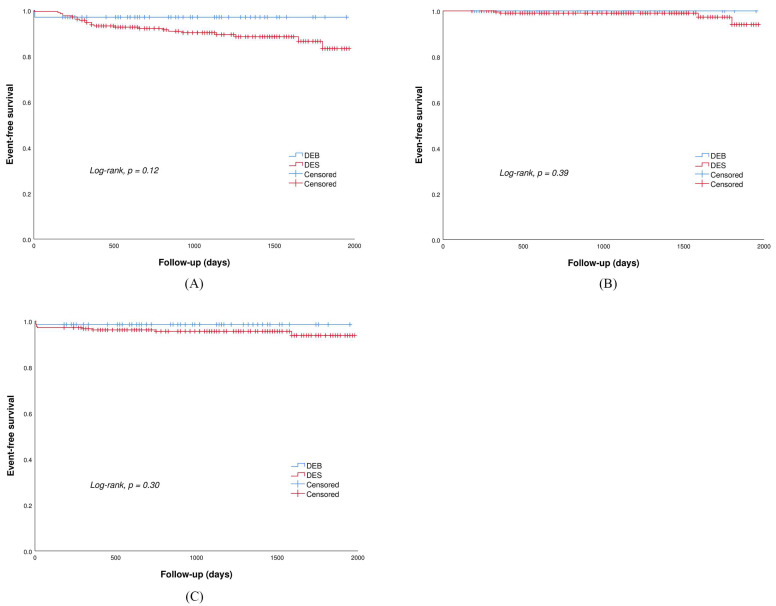
Kaplan–Meier survival curve for (**A**) DOCE: device-oriented composite endpoint; (**B**) heart failure; (**C**) major bleeding events.

Clinical follow-up data (Table 3) were obtained in all patients with a median follow-up period of 1155 days (IQR: 690, 1530 days). Despite no statistical difference, patients treated with DEBs had a trend toward a lower incidence of DOCEs (3.0% vs. 11.0%, *p* = 0.12) mainly driven by the need for TLR (3.0% vs. 9.1%, *p* = 0.19) than those treated with DESs. Specifically, in the DEB group, two patients had TLR due to coronary dissections and acute vessel closure; in the DES group, one patient had TLR due to early stent thrombosis which occurred on the third day after the index procedure, seven patients had TLR due to LST which occurred within 1 year, five patients had TLR due to VLST which occurred after 1 year, and six patients had TLR due to in-stent restenosis. Of the total of 12 patients who had LST and VLST, eight patients received DAPT at the time of the event. Additionally, no significant differences between the two groups were detected in the occurrence of cardiac death (0.0% vs. 0.5%, *p* = 0.69) and target vessel MI (0.0% vs. 1.4%, *p* = 0.40). There were no significant differences between the two groups with regard to the risk of heart failure (0.0% vs. 1.9%, *p* = 0.39) and major bleeding events (1.5% vs 4.8%, *p* = 0.30). Kaplan–Meier survival curves for the clinical outcomes are shown in Figure 2.

Multivariate regression analysis (Table 4) showed that NSTEMI (HR 3.43, 95% CI [1.36, 8.63], *p* = 0.01), Killip class ≥ 2 (HR 2.53, 95% CI [1.08, 5.95], *p* = 0.03), TIMI grade ≤ 1 (HR 4.25, 95% CI [1.25, 14.41], *p* = 0.02) and non-infarct-related artery with stenosis >70% (HR 4.02, 95% CI [1.33, 12.14], *p* = 0.01) were the independent predictors for DOCEs during the follow-up period. Additionally, DEBs were found to be associated with a trend toward a lower risk of DOCEs (HR 0.13, 95% CI [0.02, 1.05], *p* = 0.06) despite no statistical significance achieved. No independent factors for major bleeding events were observed by multivariate regression analysis (Supplementary Table S1).

In the subgroup analyses, the results of comparison between the two groups were consistent across the ten prespecified subgroups (Supplementary Figure S1). Furthermore, no significant differences between the two groups were observed with regard to clinical events occurring either within 1-year or beyond 1 year (Supplementary Tables S2 and S3).

## 4. Discussion

To the best of our knowledge, this is the first study to evaluate the safety and effectiveness of DEBs versus DESs in young patients with AMI. The present study showed that as compared with DESs, DEBs achieved a numerically lower rate of DOCEs mainly driven by the need for TLR during the mid- to long-term follow-up in the young AMI population. DEBs might be a feasible, safe, and effective alternative to DESs for the treatment of young patients with AMI.

To date, there are no specific recommendations on the management of young individuals with AMI. According to current guidelines, PCI with implantation of a permanent DES was the preferred reperfusion strategy in all patients presenting with AMI irrespective of the patient’s age [9]. However, younger age has been identified by previous studies as an independent predictor of LST and VLST after DES treatment, leading to a higher risk of repeat revascularization [11,12,19]. Consistently, the present study showed that the total rate of TLR due to LST and VLST and in-stent restenosis was relatively higher in the DES group than in the DEB group. Moreover, among the total of 12 patients who had LST and VLST, eight patients received DAPT at the time of the event. These findings suggested that younger AMI patients might have a less favorable vascular response to stent implantation. One of the possible explanations is that younger AMI patients have specific pathophysiologic characteristics and atherosclerotic plaque features compared with older cohorts with AMI [20]. Indeed, angiographic studies that used intravascular ultrasound (IVUS) and optical coherence tomography (OCT) have demonstrated that compared with older patients, younger patients are more likely to have eroded plaques, characterized by a lower percentage of dense calcium volume, necrotic core, and greater percentage of fibrous tissue volume [21,22,23]. Hu et al. suggested that eroded plaque was independently associated with less favorable healing following DES implantation at 6 months in patients with acute coronary syndrome [24]. Hong et al. found that absolute fibrous tissue volume was positively and independently associated with the development of plaque prolapse after DES implantation [25]. Similarly, Haine et al. confirmed that the fibrous tissue volume was independently related to an in-stent late luminal loss on angiography and to maximal percentage area stenosis and percentage volume intima hyperplasia on IVUS [26].

Combaret et al. proposed a two-stage management strategy in young AMI patients [27]. Based on the results of the second angiography which was performed 2 to 7 days after achieving optimal epicardial reperfusion (TIMI flow ≥ 2) by the initial strategy, patients were assigned to receive a bioresorbable vascular scaffold (BVS) in the case of stenosis greater than 70% or plaque prolapse or to receive medical treatment alone in other cases. Among the forty-five patients enrolled, only one patient in the medical group encountered recurrent MI at six months. This finding indicated that management by limiting the implantation of durable intracoronary devices was a potential treatment option in the young population. Nevertheless, the results should be interpreted with caution due to the small sample size in this “proof of concept” study, and the safety and effectiveness of bioresorbable vascular scaffolds in young AMI patients remains controversial because there was a lack of comparisons with patients managed by stenting.

DEBs are a semicompliant angioplasty balloon coated with a homogeneous distribution of the antiproliferative drug, which could be rapidly released into the target vessel wall in high concentrations [14,15]. Theoretically, DEBs could provide superiority over DESs in young AMI patients because they could avoid stent thrombosis, decrease the chronic inflammatory response, reduce delayed healing as well as maintain the coronary vasomotor response and vessel geometry with proven positive remodeling. Moreover, the ability to leave nothing behind and allow a shorter duration of DAPT might reduce mental and life stress in younger AMI patients.

Consistent with previous trials which have shown favorable clinical and angiographic outcomes of the DEB-only strategy in the general population with AMI [28,29], the present study showed that DEBs had a favorable safety and effectiveness in the treatment of the young AMI population. Indeed, in the present study, only two patients in the DEB-only arm encountered TLR during hospitalization, including one patient who required bailout stenting due to a type D dissection (National Heart, Lung, and Blood Institute classification) after DEB angioplasty and one patient who received an additional DES due to acute vessel closure which may have been caused by a delayed dissection occurring one day after the index procedure. After discharge, neither these two patients nor the remaining sixty-five patients suffered a DOCE. These findings indicated that the occurrence of coronary dissections and acute vessel closure might be the major concern of DEB use instead of implanting DESs in young AMI patients. Lin et al. demonstrated that the independent predictors of dissection after DEB treatment for patients with native coronary artery disease were women, higher DEB-to-reference vessel ratio, and longer lesion length [30]. Cortese et al. showed that patients had more severe–moderate calcification, a larger diameter of the predilation balloon, and DEBs in the dissection group than in the nondissection group [31]. In the current study, the higher DEB-to-reference vessel ratio and the longer lesion might also be the major contributors to the dissection after DEB treatment in these two patients. In addition, optimal lesion pretreatment is essential for the success of the DEB-only strategy. Iijima et al. showed that the use of a nonslip element balloon was effective for optimal lesion preparation before the use of the DEB due to its ability to reduce elastic recoil, traumatic vessel injury, and dissection [32].

Although a bailout stenting could be used as a remedial treatment for dissection, the combined use of DEBs and DESs in patients with dissection remains a matter of debate. Mitomo et al. found that the TLR rate at 2 years was up to 14.5% in patients receiving bailout stenting with DESs due to suboptimal DEB results [33]. Additionally, the second stent placement may also be psychologically stressful for younger patients. Therefore, it would be better to avoid bailout stenting as far as possible. In fact, it is sometimes possible to overestimate and underestimate the severity of the dissection using angiographic findings alone [34,35]. Spiral longitudinal dissection is usually diagnosed as type D dissection on angiography, whereas IVUS or OCT sometimes reveals less severe dissection. Conversely, hematoma as the major cause of acute occlusion sometimes cannot be identified on angiography but is clearly observed on IVUS or OCT. Intriguingly, a recent study conducted by Yamamot et al. showed that the dissection index, a new dissection grading system according to intravascular imaging findings which indicated the extent of coronary dissection after DEB treatment, was positively related to both chronic lumen and vessel enlargement [36]. A possible explanation was that dissection could help transmural diffusion of the drug and additionally force paclitaxel to be delivered in high doses near the adventitia, such as in intrapericardial paclitaxel delivery. Evidence showed that intrapericardial delivery of paclitaxel in large doses could lead to an increase in vessel enlargement and a decrease in neointimal mass by apoptotic cells. Therefore, to precisely detect the severity of dissection and optimal treatment for coronary dissection and the related prognosis after DEB angioplasty for younger AMI patients, further larger trials using IVUS or OCT should be conducted.

## 5. Limitations

The present study had several important limitations. First, it was a retrospective, single-center study with observational analysis; thus, the inherent biases cannot be completely avoided. Second, this study might be underpowered to detect statistical significance between groups due to the small sample size, especially for the DEB group. Moreover, meaningful subgroup analysis according to gender could not be performed given the limited number of patients in the female group. Third, angiographic or intravascular ultrasound follow-up could provide additional insights into the assessment of DEBs. However, due to the inherent limitations of the study design, we were unable to obtain follow-up angiographic data in all study populations. Likewise, comparative data on the prevalence of stress disorder between the two groups were also unavailable. Future prospective studies designed to assess coronary angiographic outcomes and mental health in these particular populations are required.

## 6. Conclusions

The present study demonstrated that for the young AMI population, DEBs could achieve a numerically lower rate of DOCEs mainly driven by the need for TLR compared with DESs. DEBs might be a feasible, safe, and effective treatment alternative for young patients with AMI. Further large-scale, randomized controlled trials are required to prove the benefits of the DEB strategy in this setting.

## Figures and Tables

**Table 1:** Baseline and clinical characteristics of patients.

Characteristic	Overall (*n* = 276)	DEB (*n* = 67)	DES (*n* = 209)	*p* Value
Age, years	39.3 ± 4.1	38.7 ± 4.5	39.4 ± 4.0	0.23
Male gender	262 (94.9)	66 (98.5)	196 (93.8)	0.20
BMI, kg/m^2^	27.8 ± 4.0	27.9 ± 3.4	27.7 ± 4.2	0.73
Overweight	212 (76.8)	52 (77.6)	160 (76.6)	0.86
Hypertension	126 (45.7)	31 (46.3)	95 (45.5)	0.90
Diabetes mellitus	51 (18.5)	15 (22.4)	36 (17.2)	0.34
Hyperlipidemia	62 (22.5)	18 (26.9)	44 (21.1)	0.32
Asthma	5 (1.8)	2 (3.0)	3 (1.4)	0.60
Chronic kidney disease	8 (2.9)	2 (3.0)	6 (2.9)	1.00
Stroke	11 (4.0)	3 (4.5)	8 (3.8)	0.73
Current smoking	226 (81.9)	53 (79.1)	173 (82.8)	0.50
Prior PCI and CABG	4 (1.4)	2 (3.0)	2 (1.0)	0.25
Family history of coronary artery disease	122 (44.2)	27 (40.3)	95 (45.5)	0.46
Clinical presentation at admission				
STEMI	172 (62.3)	32 (47.8)	140 (67.0)	0.01
NSTEMI	104 (37.7)	35 (52.3)	69 (33.3)	0.01
Admission hemodynamics				
Systolic blood pressure, mmHg	128.8 ± 19.7	130.1 ± 19.7	128.3 ± 19.7	0.52
Diastolic blood pressure, mmHg	79.0 ± 14.6	80.7 ± 14.4	78.5 ± 14.6	0.29
Heart rate, bpm	80.8 ± 16.6	82.1 ± 12.4	80.4 ± 17.8	0.46
Killip class at admission				
1	194 (70.3)	52 (77.6)	142 (67.9)	0.13
2–3	82 (29.7)	15 (22.4)	67 (32.1)	0.13
LVEF, %	59.7 ± 10.4	58.8 ± 10.3	60.0 ± 10.4	0.39
Laboratory findings at admission				
TNI, ng/mL	16.16 (1.44, 62.42)	10.11 (0.61, 39.62)	16.81 (1.55, 64.90)	0.37
CKMB, ng/mL	18.89 (2.40, 84.70)	13.70 (1.80, 75.70)	21.95 (2.68, 93.73)	0.20
BNP, pg/mL	70.00 (33.00, 164.50)	66.0 (41.0, 174.0)	72.5 (31.25, 159.50)	0.75
ALB, g/L	43.22 ± 5.26	42.77 ± 4.45	43.36 ± 5.49	0.42
GLB, g/L	27.38 ± 4.75	26.62 ± 4.95	27.62 ± 4.67	0.13
TC, mmol/L	5.20 ± 1.31	5.32 ± 1.55	5.16 ± 1.23	0.40
TG, mmol/L	2.82 ± 2.45	3.10 ± 2.79	2.73 ± 2.33	0.28
HDL-C, mmol/L	0.93 ± 0.22	0.90 ± 0.19	0.95 ± 0.23	0.10
LDL-C, mmol/L	3.43 ± 1.28	3.60 ± 1.57	3.37 ± 1.17	0.21
LPA, mg/dL	21.71 ± 21.11	24.06 ± 23.88	20.96 ± 20.15	0.30
GLU, mmol/L	8.04 ± 3.92	8.08 ± 3.97	8.03 ± 3.91	0.93
CR, umol/L	72.20 (64.65, 81.40)	72.80 (62.7, 79.9)	72.20 (65.4, 81.63)	0.80
WBC, 10^9^/L	10.86 ± 3.72	10.36 ± 3.16	11.02 ± 3.87	0.21
HGB, g/L	151.06 ± 20.08	151.27 ± 27.49	151.00 ± 17.12	0.92
PLT, 10^9^/L	249.07 ± 72.73	250.21 ± 56.77	248.71 ± 77.27	0.88
D-dimer, mg/L	0.19 (0.13, 0.32)	0.19 (0.12, 0.41)	0.19 (0.14, 0.31)	0.66
FIB, mg/dL	312.95 ± 109.22	335.57 ± 125.97	305.70 ± 102.57	0.08

BMI: body mass index; TNI: troponin I; CK-MB: creatine kinase isoenzyme MB; BNP: brain-type natriuretic peptide; LAC: lactic acid; TC: total cholesterol; TG: triglyceride; HDL: high-density lipoprotein cholesterol; LDL: low-density lipoprotein cholesterol; UA: uric acid; CR: creatinine; WBC: white blood cells; HGB: hemoglobin; PLT: platelet; FIB: fibrinogen; DEB: drug-eluting balloon; DES: drug-eluting stent.

**Table 2:** Procedural and angiographic characteristics of patients.

Characteristic	Overall (*n* = 276)	DEB Group (*n* = 67)	DES Group (*n* = 209)	*p* Value
Infarct-related artery				
Left anterior descending coronary artery	115 (41.7)	19 (28.4)	96 (45.9)	0.01
Right coronary artery	97 (35.1)	19 (28.4)	78 (37.3)	0.18
Left circumflex coronary artery	64 (23.2)	29 (43.3)	35 (16.7)	<0.01
TIMI flow grade before PCI				0.44
0–1	183 (66.3)	47 (70.1)	136 (65.1)	
2–3	93 (33.7)	20 (29.9)	73 (34.9)	
Device characteristics				
Total number	1.0 (1.0, 2.0)	1.0 (1.0, 1.0)	2.0 (1.0, 2.0)	<0.01
Total length, mm	29.5 (22.00, 40.75)	26.00 (20.00, 30.00)	30.00 (23.5, 46.00)	<0.01
Minimum diameter, mm	3.00 (2.75, 3.50)	2.75 (2.00, 3.00)	3.00 (3.00, 3.50)	<0.01
Maximal pressure, atm	10.28 ± 1.86	9.31 ± 1.84	10.58 ± 1.76	<0.01
Inflation time, sec	5 (5, 20)	60 (60, 60)	5 (5, 10)	<0.01
Thrombus aspiration	54 (19.6)	6 (9.0)	48 (23.0)	0.01
Intra-aortic balloon pump	24 (8.7)	7 (10.4)	17 (8.1)	0.56
Glycoprotein IIb/IIIa therapy	72 (26.1)	15 (22.4)	57 (27.3)	0.92
TIMI flow grade 3 post-PCI	273 (98.9)	66 (98.5)	207 (99.0)	0.57
Non-infarct-related artery with stenosis > 70%	174 (63.0)	47 (70.1)	127 (60.8)	0.17
Immediate complete revascularization (*n* = 174)	54 (31.03)	25 (53.2)	29 (22.8)	<0.01
Medication at discharge				
Aspirin	276 (100.0)	67 (100.0)	207 (100.0)	1.00
Clopidogrel	218 (79.0)	56 (83.6)	162 (77.5)	0.29
Ticagrelor	58 (21.0)	11 (16.4)	47 (22.5)	0.29
Statins	245 (88.8)	60 (89.6)	185 (88.5)	0.82
Beta-blockers	207 (75.0)	49 (73.1)	158 (75.6)	0.69
ACEI/ARB	161 (58.3)	37 (55.2)	124 (59.3)	0.55
DAPT duration	12 (9, 12)	6 (5, 6)	12 (12, 12)	< 0.01

TIMI: thrombolysis in myocardial infarction; PCI: percutaneous coronary intervention; ACEI/ARB: angiotensin-converting enzyme inhibitors/angiotensinogen type II receptor blockers; DEB: drug-eluting balloon; DES: drug-eluting stent.

**Table 3:** Clinical outcomes after the index procedure.

	Overall (*n* = 276)	DEB Group (*n* = 67)	DES Group (*n* = 209)	Log-Rank *p* Value
DOCE	25 (9.1)	2 (3.0)	23 (11.0)	0.12
Cardiac death	1 (0.4)	0 (0.0)	1 (0.5)	0.69
Target vessel MI	3 (1.1)	0 (0.0)	3 (1.4)	0.40
TLR	21 (7.6)	2 (3.0)	19 (9.1)	0.19
Major bleeding events	11 (4.0)	1 (1.5)	10 (4.8)	0.30
Heart failure	4 (1.4)	0 (0.0)	4 (1.9)	0.39

DOCE: device-oriented composite endpoint; MI: myocardial infarction; TLR: target lesion revascularization; DEB: drug-eluting balloon; DES: drug-eluting stent.

**Table 4:** Multivariate regression analysis of the predictors for DOCE.

Variables	HR	95% CI	*p* Value
Age	0.942	0.856, 1.037	0.223
DEB	0.130	0.016, 1.053	0.056
NSTEMI	3.430	1.364, 8.626	0.009
Killip class ≥ 2	2.532	1.077, 5.953	0.033
Left anterior descending artery	0.862	0.370, 2.006	0.730
TIMI grade ≤ 1	4.250	1.254, 14.406	0.020
DEB or DES number	1.189	0.336, 4.213	0.788
DEB or DES diameter	1.080	0.785, 1.485	0.637
DEB or DES length	0.993	0.951, 1.037	0.754
Thrombus aspiration	1.494	0.582, 3.837	0.404
Glycoprotein IIb/IIIa therapy	1.743	0.749, 4.055	0.197
NIRA > 70%	4.018	1.329, 12.142	0.014
DAPT duration	0.923	0.755, 1.129	0.436

DOCE: device-oriented composite endpoint; DEB: drug-eluting balloon; DES: drug-eluting stent; NSTEMI: non-ST-segment elevation myocardial infarction; TIMI: thrombolysis in myocardial infarction; NIRA: non-infarct-related artery with stenosis; DAPT: dual antiplatelet therapy.

## Data Availability

The data that support the findings of this study are available from the corresponding author, Le-Feng Wang, upon reasonable request.

## Supplementary Materials

The following supporting information can be downloaded at: https://www.mdpi.com/article/10.3390/jcdd10010029/s1, Figure S1: Influence of the 10 pre-specified variables on the risk of MACE between the two treatment groups; Table S1: Multivariate regression analysis of the predictors for major bleeding events; Table S2: Clinical outcomes during 1-year of follow-up; Table S3: Clinical outcomes beyond 1-year of follow-up.
