# Supplementary material for: Comparisons of Drug-Eluting Balloon versus Drug-Eluting Stent in the Treatment of Young Patients with Acute Myocardial Infarction
Source: J Cardiovasc Dev Dis. 2023 Jan 13;10(1):29. doi: 10.3390/jcdd10010029 (PMC9865202; doi:10.3390/jcdd10010029)
Supplement: Supplementary file 1 [file jcdd-10-00029-s001.zip › jcdd-2115860-supplementary.pdf]

Subgroup	DEB	DES	HR	95% CI	P value	Interacion P
Age ≤ 40 years	2/38 (5.3%)	14/114 (12.3%)	0.47	0.11, 2.08	0.32	0.3
Age > 40 years	0/29 (0.0%)	9/95 (9.5%)	0.04	0.00, 84.78	0.4	
STEMI	2/32 (6.3%)	11/140 (7.9%)	1.12	0.25, 5.07	0.89	0.96
NSTEMI	0/35 (0.0%)	12/69 (17.4%)	0.03	0.00, 4.30	0.16	
LAD	1/19 (5.3%)	10/96 (10.4%)	0.75	0.10, 5.93	0.79	0.14
No LAD	1/48 (2.1%)	13/113 (11.5%)	0.22	0.03, 1.67	0.14	
Killip class 0-1	1/52 (1.9%)	11/142 (7.7%)	0.28	0.04, 2.17	0.22	0.12
Killip class 2-3	1/15 (6.7%)	12/67 (17.9%)	0.61	0.08, 4.81	0.64	
TIMI grade pre-PCI 0-1	2/47 (4.3%)	19/136 (14.0%)	0.37	0.09, 1.59	0.18	0.39
TIMI grade pre-PCI 2-3	0/20 (0.0%)	4/73 (5.5%)	0.04	0.00, 2862.85	0.57	
Device diameter < 3 mm	1/43 (2.3%)	5/47 (10.6%)	0.29	0.03, 2.53	0.26	0.52
Device diameter ≥3 mm	1/24 (4.2%)	18/162 (11.1%)	0.45	0.06, 3.35	0.43	
Device length < 30 mm	0/40 (0.0%)	7/98 (7.1%)	0.03	0.00, 48.93	0.36	0.89
Device length ≥ 30 mm	2/27 (7.4%)	16/111 (14.4%)	0.59	0.14, 2.58	0.49	
Thrombosis aspiration	0/6 (0.0%)	8/48 (16.7%)	0.04	0.00, 5330.33	0.6	0.18
No thrombosis aspiration	2/61 (3.3%)	15/161 (9.3%)	0.4	0.09, 1.77	0.23	
GP IIb/IIIa inhibitors	1/15 (6.7%)	9/57 (15.8%)	0.74	0.09, 6.12	0.78	0.12
No GP IIb/IIIa inhibitors	1/52 (1.9%)	14/152 (9.2%)	0.24	0.03, 1.80	0.17	
NIRA ≥ 70%	2/47 (4.3%)	19/127 (15.0%)	0.34	0.08, 1.48	0.15	0.39
NIRA < 70%	0/20 (0.0%)	4/82 (4.9%)	0.04	0.00, 4189.82	0.58	

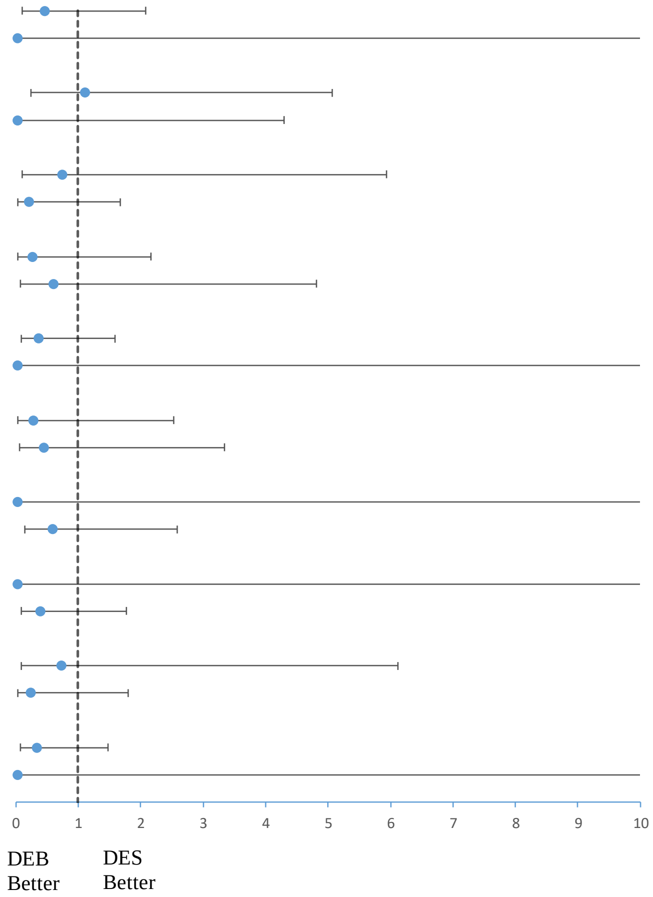

Supplementary Figure S1. Influence of the 10 pre-specified variables on the risk of DOCE between the two treatment groups. All interaction tests were negative. STEMI: ST-segment elevation myocardial infarction; NSTEMI: non-ST-segment elevation myocardial infarction; LAD: left anterior descending; TIMI: thrombolysis in myocardial infarction; GP: Glycoprotein; NIRA: non-infarct related artery.

Supplementary Table S1. Multivariate regression analysis of the predictors for major bleeding events.

Variables	HR	95% CI	*p* value
Age	0.916	0.799, 1.050	0.207
DEB	0.855	0.054, 13.469	0.912
NSTEMI	0.240	0.045, 1.281	0.095
Killip class ≥ 2	0.529	0.107, 2.625	0.436
Left anterior descending artery	0.607	0.165, 2.237	0.454
TIMI grade ≤ 1	0.407	0.102, 1.627	0.203
DEB or DES number	1.374	0.210, 8.983	0.740
DEB or DES diameter	1.092	0.783, 1.522	0.604
DEB or DES length	0.996	0.931, 1.065	0.902
Thrombus aspiration	0.820	0.151, 4.444	0.818
Glycoprotein IIb/IIIa therapy	0.319	0.038, 2.692	0.293
NIRA > 70%	0.375	0.100, 1.403	0.145
DAPT duration	1.116	0.821, 1.517	0.485

DEB: drug-eluting balloon; DES: drug-eluting stent; NSTEMI: non-ST-segment elevation myocardial infarction; TIMI: thrombolysis in myocardial infarction; NIRA: non-infarct-related artery with stenosis; DAPT: dual antiplatelet therapy.

Supplementary Table S2. Clinical outcomes during 1-year of follow-up

	Overall (*n* = 276)	DEB group (*n* = 67)	DES group (*n* = 209)	*p* value
DOCE	14 (5.1)	2 (3.0)	12 (5.7)	0.529
Cardiac death	0 (0.0)	0 (0.0)	0 (0.0)	1.000
Target vessel MI	2 (0.7)	0 (0.0)	2 (1.0)	1.000
TLR	12 (4.3)	2 (3.0)	10 (4.8)	0.736
Major bleeding events	9 (3.3)	1 (1.5)	8 (3.8)	0.692
Heart failure	2 (0.7)	0 (0.0)	2 (1.0)	1.000

DOCE: device-oriented composite endpoint; MI: myocardial infarction; TLR: target lesion re-vascularization; DEB: drug-eluting balloon; DES: drug-eluting stent.

Supplementary Table S3. Clinical outcomes beyond 1-year of follow-up

	Overall (*n* = 276)	DEB group (*n* = 67)	DES group (*n* = 209)	*p* value
DOCE	11 (4.0)	0 (0.0)	11 (5.3)	0.071
Cardiac death	1 (0.4)	0 (0.0)	1 (0.5)	1.000
Target vessel MI	1 (0.4)	0 (0.0)	1 (0.5)	1.000
TLR	9 (3.3)	0 (0.0)	9 (4.3)	0.119
Major bleeding events	2 (0.7)	0 (0.0)	2 (1.0)	1.000
Heart failure	2 (0.7)	0 (0.0)	2 (1.0)	1.000

DOCE: device-oriented composite endpoint; MI: myocardial infarction; TLR: target lesion re-vascularization; DEB: drug-eluting balloon; DES: drug-eluting stent.
